# Exploring quality improvement processes for psychotropic medication use in Australian residential aged care homes: a qualitative study

**DOI:** 10.1080/20523211.2025.2557873

**Published:** 2025-09-22

**Authors:** Shakti Shrestha, Amanda J. Cross, Michelle Steeper, Nazanin Falconer, Laurie Buys, Carmela Lagasca, Angelita Martini, Dayna Cenin, Nancy Ochieng, Francesca Glamorgan, J. Simon Bell, Adam La Caze

**Affiliations:** a School of Pharmacy and Pharmaceutical Sciences, Faculty of Health, Medicine and Behavioural Sciences, The University of Queensland, Brisbane, Australia; b Centre for Medicine Use and Safety, Faculty of Pharmacy and Pharmaceutical Sciences, Monash University, Parkville, Australia; c School of Public Health and Preventive Medicine, Faculty of Medicine, Nursing and Health Sciences, Monash University, Melbourne, Australia; d Faculty of Health Sciences, Australian Catholic University, Brisbane, Australia; e Anglicare Southern Queensland, Brisbane, Australia; f Calvary Health Care, Waratah, Australia; g The University of Western Australia, Perth, Australia; h Brightwater Research Centre, Brightwater Care Group, Inglewood, Australia; i Lifeview Corporate Lifeview Pty Ltd., Carnegie, Australia; j Montefiore, Randwick, Australia

**Keywords:** Long-term care, residential facilities, psychotropic drugs, dementia, quality improvement, learning health system

## Abstract

**Background:**

Recent regulatory changes in Australia have emphasised system-level approaches to ensure appropriate psychotropic medication use in residential aged care homes. This study explored quality improvement processes related to psychotropic medication use in Australian residential aged care homes.

**Methods:**

This qualitative study used in-depth semi-structured interviews with a maximum variation sample of key stakeholders involved in psychotropic medication use at organisations operating facilities in metropolitan and regional areas in four Australian states. The interviews were transcribed verbatim and thematically analysed using both inductive and deductive approaches by two researchers using a framework developed for learning health systems.

**Results:**

Stakeholders (n = 33) included nurses, occupational therapists, pharmacists, medical practitioners, residents and caregivers. Identified themes were (i) regulation was driving change in organisational policies and procedures, and (ii) aged care organisations were enhancing quality improvement systems for psychotropic medications. Many of the requirements of successful healthcare quality improvement systems were present within the aged care organisations, including alignment of core values and presence of key ethical, legal and policy infrastructure. There are opportunities for better use of clinical data to improve care, especially in terms of learning from the data and implementing tailored change. The challenges identified by participants included navigating the perceived tension between compliance and quality, and aligning the goals and processes of all health professionals.

**Conclusions:**

Recent changes in policies, procedures and infrastructure have provided clearer oversight of psychotropic medication use. Individual and system approaches to psychotropic medication use in aged care have shifted. Key opportunities for improving use of psychotropic medications within aged care organisations include improving the capacity to use local data to improve care and building interdisciplinary teams to facilitate collaborative care.

## Background

In the Australian regulatory context, any medication capable of affecting the mind, emotions and behaviour is defined as psychotropic medication (Australian Government, 2022a). Antipsychotics, antidepressants and benzodiazepines are frequently prescribed psychotropic medications for managing changed behaviours in people living with dementia (Brimelow et al., 2019, 2023; Harrison et al., 2020; Westbury et al., 2019). Changed behaviours in dementia, also known as the behavioural and psychological symptoms of dementia (BPSD), may include agitation, aggression, verbal behaviour, wandering, inappropriate sexual behaviour and resistance to care. Evidence for the effectiveness of psychotropic medications for changed behaviour is limited (Dudas et al., 2018; Farina et al., 2017; Seitz et al., 2011), and long-term use poses a risk of harm (Dyer et al., 2018; Schneider et al., 2006; Wu et al., 2009). Clinical practice guidelines recommend tailored non-pharmacological behaviour support strategies as the primary approach for managing changed behaviour (National Institute for Clinical Excellence, 2018; RACGP, 2023). A wide range of strategies to support appropriate psychotropic medication use in residential aged care homes have been employed at the regulatory, organisational and clinical levels (Aged Care Quality and Safety Commission, 2024; Australian Government, 2021, 2024; Langford et al., 2020; Royal Commission, 2019). Nevertheless, ensuring appropriate use of psychotropic medications in residential aged care homes remain a challenge (Langford et al., 2020; Royal Commission, 2019).

Aged care in Australia includes a variety of home care/home support packages and residential aged care. Residential aged care homes in Australia provide support to people who need a higher level of care than can be provided at home. In 2024, there were 736 approved residential aged care providers in Australia supplying approximately 240,000 places (Department of Health and Aged Care, 2024). Residential aged care homes in Australia are managed by government, not-for-profit and private organisations. Most prescribing within residential aged care homes is provided by visiting general practitioners. People living in residential aged care homes may also be referred to specialist care (including geriatricians and psychiatrists) and some facilities employ on-site nurse practitioners (Sluggett et al., 2017). Medications are dispensed by off-site community pharmacies. The estimated prevalence of dementia in Australia is 15 per 1000 Australians and 84 per 1000 Australians aged 65 and over (Australian Institute of Health and Welfare, 2024). Approximately half of the people living in permanent residential aged are estimated to be living with dementia (Australian Institute of Health and Welfare, 2024).

In March 2021 the Royal Commission into Aged Care Quality and Safety highlighted the need for improved communication, documentation and quality care processes surrounding psychotropic use in Australia (Australian Government, 2021). The regulatory changes implemented following the Royal Commission include a formal definition of restrictive practices (including chemical restraint), stricter criteria regarding the use of restrictive practices, additional requirements regarding consent as well as documentation and reporting requirements (Aged Care Quality and Safety Commission, 2024). The key components of these changes are outlined in Supplemental Material 1. Within this context, ‘restrictive practice’ is defined as any action that restricts the rights or freedom of movement of a care recipient (Australian Government, 2022a). ‘Chemical restraint’ is a form of restrictive practice that refers to the use of medication or a chemical substance for the primary purpose of influencing a care recipient’s behaviour (some exceptions are permitted, such as when the medication is used in the treatment of a diagnosed mental disorder or as part of end of life care) (Australian Government, 2024). The regulatory changes provide additional responsibilities to health professionals (especially nursing and medical staff) in terms of providing and documenting care and residential aged care providers in terms of ensuring the care provided within the facility complies with the regulatory requirements. To facilitate compliance with requirements in relation to chemical restraint, residential aged care organisations were advised to maintain a ‘psychotropic register’ containing details of all residents receiving psychotropic medications (Aged Care Quality and Safety Commission, 2022).

These Australian regulatory changes are supported by the National Aged Care Mandatory Quality Indicator Program (which includes and indicator regarding antipsychotic use) and a digitisation program for aged care health systems (Australian Government, 2023; Bell et al., 2022). Digital prescribing and medication management systems are being progressively implemented in residential aged care homes (Seaman et al., 2021). There are also significant ongoing efforts to support medication use within residential aged care homes such as federally funded residential medication management reviews (RMMR), educational and quality improvement activities funded as part of the Quality Use of Medicines Program, and medication advisory committees (Chen et al., 2019; Picton et al., 2020). Lastly, funding for embedded onsite pharmacists at residential aged care homes was announced in March 2022 (Cross et al., 2022, 2024) and Australia’s new *Clinical Practice Guidelines for the Appropriate Use of Psychotropic Medications in People Living with Dementia and in Residential Aged Care* (new psychotropic guidelines) were approved by the National Health and Medical Research Council in April 2023 (Bell et al., 2022).

While the overall medication management process in Australian residential aged care homes has been described (Sluggett et al., 2017), there is limited literature on the impact of the significant regulatory changes in relation to management of change behaviour and the use of psychotropic medications within these facilities. This study sought to address this gap by exploring quality improvement processes related to psychotropic medication use in Australian residential aged care homes following the regulatory changes.

## Methods

### Study design and setting

This study employed a qualitative study design. The consolidated criteria for reporting qualitative research (COREQ) was used to ensure the quality of reporting (Booth et al., 2014) (Supplemental Material 1). The study was conducted with a convenience sample of four residential aged care organisations from across Australian states (Queensland, New South Wales, Victoria and Western Australia). Each organisation operated aged care homes in metropolitan and regional areas (Supplemental Material 3) and were included in the study on the basis of participating in a broader project seeking to translate the new psychotropic guidelines into practice (Bell et al., 2024). Aged care organisations are responsible for ensuring individual facilities comply with regulations regarding the quality and safety of care. Staff in leadership roles in clinical governance tended to work across facilities (e.g. Clinical Governance Lead, Manager of Clinical Services) within the organisation and support staff in leaderships roles within facilities (e.g. Facility Managers, Care Coordinators).

### Participants

Participants were key stakeholders of aged care homes and were recruited in collaboration with the four participating aged care organisations. A maximum variation sample was sought in terms of different levels of employees within the organisation (specifically the inclusion of a facility manager, quality manager and registered nurse), visiting healthcare professionals (specifically: general practitioner, geriatrician, community pharmacist, clinical services pharmacist), and residents and family/informal caregivers involved in quality improvement processes related to psychotropic medications (Supplemental Material 4). The organisation sent an invitation to potential participants for voluntary participation in the study. Additional follow up was required to ensure participation from more than one general practitioner and to recruit a geriatrician. Prospective participants were contacted by the research team to arrange for one-on-one semi-structured interviews over Zoom or Microsoft Teams.

### Data collection

Interviews were conducted between August to December 2022 by academic pharmacists with experience in aged care and qualitative research (AL, SS). The researchers were unknown to the participants apart from one pre-existing professional relationship. All interviews were recorded (audiovisual) and transcribed verbatim. An interview guide (Supplemental Material 5) was developed by the research team and refined through discussion with two experts in psychotropic medication use in aged care. Specific wording used within the guide was modified to ensure it was appropriate for healthcare providers/facility staff and residents/carers. Participant information regarding their roles and profession was collected. An interview summary was provided to each participant to provide an opportunity for member checking.

### Data analysis

Transcripts were thematically analysed (Braun & Clarke, 2006). A deductive analysis was conducted using a framework for learning health systems (Menear et al., 2019), which was supplemented by an inductive analysis seeking to identify any additional data-derived codes and themes. Deductive analysis examined policies and practices in relation to psychotropic medications within each organisation for consistency with the components of best-practice quality improvement systems as informed by the framework for learning health systems (Menear et al., 2019). Two investigators (AL, SS) independently coded transcripts using NVivo Qualitative Data Analysis Software v.1.6.1 (QSR International, USA), ensured coding consistency and developed inductive coding scheme (Supplemental Material 6) through regular discussion with the wider team starting early in the analysis and continuing throughout as consensus developed regarding the identified themes.

A learning health system is a ‘system designed to generate and apply the best evidence for collaborative healthcare choices for each patient and organisation; to drive the process of discovery as a natural outgrowth of patient care; and to ensure innovation, quality, safety, and value in health care’ (Institute of Medicine, 2011). Menear and colleagues’ framework for learning health systems provided an overall theoretical framework for exploring the people, processes and infrastructure related to the quality improvement system in place within the participating aged care organisations seeking to ensure appropriate psychotropic medication use (Menear et al., 2019). The framework identifies the core values, key pillars (infrastructure), processes and outcomes that support organisations to achieve high quality and high value care.

In terms of reflexivity, AL and SS are male and have expertise in medicine management and the quality use of psychotropic medications in people living with dementia. AL trained and has worked as a pharmacist and academic primarily within Australia. SS trained and worked as a pharmacist, carer and academic in Nepal, Great Britain and Australia. Neither AL or SS worked within aged care organisations during the implementation of the regulatory changes relating to psychotropic medications. AL and SS brought a recognition of the challenges of implementing change in quality improvement systems to the analysis and a professional curiosity to ways in which the people, processes and infrastructure involved in ensuring quality use of psychotropics within aged care organisations were adapting to change. The broader research team includes researchers with expertise in medication management in aged care and pharmacy practice (JSB, AJC, NF, MS), social scientists with a focus on gerontology (LB), and leaders with a focus on quality and safety from the participating aged care organisations (CL, AM, DC, NO, FG).

### Ethics

This study was approved by the University of Queensland Human Research Ethics Committee-HABS LNR (2022/HE001147) on July 19, 2022. Prior to conducting the study, an approval was sought from each participating aged care provider organisation and a written consent was obtained from each participant.

## Results

Thirty-three people participated in the interviews with 4–12 from each organisation. There were five consumers (two residents, three caregivers), 10 visiting health service providers (pharmacists, general practitioners, geriatrician) and 18 staff members (in different roles and different levels of the organisations) (Table 1). The average interview duration was 28 minutes.

**Table 1.** Participant profession and role descriptor.

Profession	Role descriptor	Participants (n)
Nurse (n = 15)	Care manager	8
	Clinical governance	3
	Nurse	1
	Nurse practitioner	2
	Research and innovation	1
Pharmacist (n = 8)	Clinical governance	1
	Clinical pharmacist	5
	Embedded pharmacist	1
	Pharmacist	1
Consumer (n = 5)	Resident	2
	Resident family member	3
Medical Practitioner (n = 3)	General practitioner	2
	Geriatrician	1
Occupational therapist (n = 2)	Dementia care	2
Total		**33**

**Figure 1.**
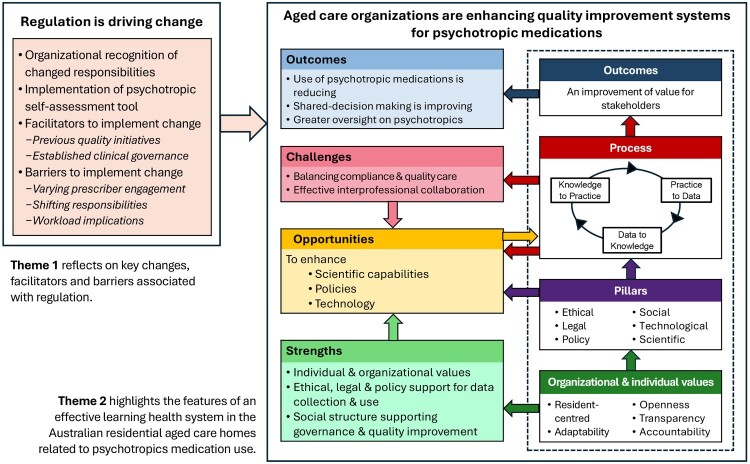
Summary of key findings. The participating aged care organisations are a developing learning health system.

Overall participants felt that the regulatory changes in relation to use of psychotropic medications has improved oversight of these medicines within the sector and have led to a greater awareness of regulation requirements and harms of psychotropic medications. Participants recognised the work involved in implementing regulatory changes and identified a tension that can arise meeting perceived regulatory requirements and providing high quality care. In terms of quality improvement processes, participants identified similarities across organisations in terms of care delivery, processes for quality improvement in relation to psychotropic medications, and challenges in implementing change across the organisations. There were considerable differences between organisations in terms of systems supporting delivery and documentation of care. Each organisation used different tools with varying levels of digitisation. There were also differences in terms of organisational culture and history in relation to managing changed behaviours, which influenced the kinds of strategies employed to meet new regulatory requirements (Figure 1). Two broad themes – ‘Regulation is driving change’ and ‘Aged care organisations are enhancing quality improvement systems for psychotropic medications’ – were identified.

### Regulation is driving change (Theme 1)

All participants reflected on the significant amount of change over the previous two years. The immediate driver for this change was regulatory (see Supplemental Material 1 for more information about regulatory changes). This regulatory change was associated with an internal recognition within aged care organisations of their responsibilities in relation to the management of changed behaviour in residents with cognitive impairment, and the development of systems and processes to collect and review information on use of psychotropic medications. Example quotes for Theme 1 are presented in Table 2 and discussed below.

**Table 2.** Supporting quotes for Theme 1 (Regulation is driving change).

Theme 1 components	Supporting quotes
Organisational recognition of changed responsibilities	*Clinical governance/Nurse/0301:* Yeah. So a part of what we've done now, so the now state, the discreet change that's mucked everybody up is – it was handed back to the sites to start making some of the determination around if there's an indication for that medication or not. So that's now handed back to the RNs *[Registered Nurses]* at site to do that first review. If they're not sure or it's not clear, then we hand it to the prescriber. So we do that first pass just using MIMS *[Monthly Index of Medical Specialties]*. Or using MIMS and the diagnoses we have available.
Implementation of psychotropic self-assessment tool	*Nurse practitioner/Nurse/0204:* For the psychotropic self-assessment tool, which is automatically generated. So that was a big project we did in July 2021. So as the commission requirement to have the self-assessment tool, it was all in Excel. And in July 2021, we transition into an online platform … . So that was a huge project that we did. So every time when a GP *[General Practitioner]* prescribes a psychotropic medication or any changes happens in that prescription, it goes automatically into the online system.
	*Care manager/Nurse/0302:* The challenges of the live risk register is when the Commission comes, it's – it gets updated once a week by me. But if a medication’s been missed or not picked up during medication rounds, then it does make it very, very hard to continue. It's a very manual task. There's nothing connected from our pharmacy system to our system that we use on the risk register. *Care manager/Nurse/0403:* But yeah, there's a lot of time spent on that psychotropic register since it's been implemented. … And you know you can't really avoid it … . There's so much time and so many days spent on just looking at making sure we've got a log of it that sometimes we're not looking around the quality of what's happening with those medications.
Facilitators to implement change – Previous quality initiatives	*Care manager/Nurse/0404:* I’ve run dementia services … So I start by looking at the incidents, and then I look at the time where the highest number of incidents are, where the highest number of behaviours are, and then I also look at where is the highest PRN *[pro re nata or when required]* psychotropic usage. And I also look at, okay, what days of the week … you have to depend on your stats and then you have to look into the time and then put interventions there and then – and I also did the Plan-Do-Check-Act cycle, PDSA *[Plan-Do-Study-Act]* cycle to see when I do the interventions, is that working after a few weeks?
	*Pharmacist/0206:* … even before their quality indicators were telling us, they were even looking at things like the use of antiepileptics used for mood stabilisation. [A leading clinician has] always had his influence in there. I remember at one MAC *[Medication Advisory Committee]* meeting the discussion was that he thought that, perhaps, their use of antiepileptics inappropriately for behavioural, and they were trying to look at that. But that was two or three years ago.
Facilitators to implement change – Established clinical governance	*Care manager/Nurse/0403:* Once a month, we have a continuous quality improvement meeting, CQI meeting. So yeah. Yeah, that talks about all areas within the facility, so hospitality improvement, things like that, but there is a big focus obviously on the clinical side of things and how that can be improved.
	*Care manager/Nurse/0406:* So we update it according to that and we also discuss it in our three monthly MAC meetings with the pharmacy as well, our ratios of psychotropics and polypharmacy and all that key areas.
Barriers to implement change – Inconsistent prescriber engagement	*Nurse practitioner/Nurse/0303:* The GPs are hit and miss, so some are great. Others rarely want to do any of the forms. They see it as a form filling rather than reviewing the medications.
Barriers to implement change – Shifting responsibilities	*Nurse practitioner/Nurse/0303:* And the other thing I notice some GPs do is they determine them as non-chemical restraints if they're prescribed for something like BPSD *[Behavioural and Psychological Symptoms in Dementia]* … So the role is really from a prescriber point of view, even though we're probably not the prescribers, but it's in lieu of the GP not being particularly on board with doing that.
Barriers to implement change – Workload implications	*General practitioner/0209:* Yeah. Random. Random drugs. I'm telling you, ‘Okay. Fine. Pregabalin, antidepressants, antipsychotics, mood stabilisers? Granted, right? I go with it, right?’ Opioids. Completely different group, but also highly, highly granted. But Maxolon, Stemetil, Motilium – what else? – PRN in case they vomit. So I have to validate the Maxolon every three months, but write the reason.

Note: Identifier – Role/Profession/Organisation and Participant Code.

#### Organisational recognition of changed responsibilities

Participants reflected on shifting responsibilities for managing changed behaviours. Previously the determination of whether a psychotropic medication was being used for changed behaviour was primarily left to prescribers. The regulatory changes prompted each organisation to change care and governance processes, which included facility staff seeking to make a determination whether a psychotropic medication was being used for changed behaviour. Prescriber input was sought when necessary. For example: ‘ … it was handed back to the sites to start making some of the determination around if there's an indication for that medication or not …  … If they're [sites are] not sure or it's not clear, then we hand it to the prescriber … [Clinical governance/Nurse/0301]’. Most participants noted this shift in responsibilities and many participants highlighted the tensions that can occur when facility staff and prescribers have different interpretations of either the regulatory requirements or the indication for a psychotropic for a specific resident.

Attitudes to the shift in responsibilities differed between the organisations. Some viewed the change in a positive light ‘ … they're [facility staff are] sort of now being accountable, to a large degree, for the use of psychotropics at facilities, and they're making sure that they are finding out that – or matching an indication for the use of any psychotropic … . [Clinical services pharmacist/Pharmacist/0304]’, while others expressed some frustration feeling that it was primarily the role of the prescriber to make this determination. ‘ … Well, they're prescribing it. They should know. And if they can't answer that question, why are we answering that question? Because they prescribed it, so it's their responsibility … [Clinical governance/Nurse/0301]’.

#### Implementation of psychotropic self-assessment tool

The Aged Care Quality and Safety Commission’s *Psychotropic self-assessment tool* was implemented in each organisation [Nurse practitioner/Nurse/0204] as a part of clinical governance processes to create a *psychotropic register*. The intention of the tool is to help organisations meet their regulatory responsibilities in terms of identifying chemical restraint, ensuring consent has been obtained and documented and behavioural support plans are in place when required. Each organisation relied on a mixture of digital and manual processes to collect the data [Care manager/Nurse/0302]. The specific processes within the organisations differed considerably due to the varied physical and digital documentation systems. The work in developing the psychotropic register was significant and tended to be iterative as advice and decisions regarding what to include changed over time.

It's much better now, but for example, in the last one year, we have changed the template three times because with the Commission [Royal Commission into Aged Care] changing the template and now including the behaviour support plan interventions and all that. So it is time-consuming when you have to start fresh and make it again. But now it's okay because it's all done and we just have to update. [Care manager/Nurse/0406]

Decision-making at each organisation was informed by a desire to minimise the risk of regulatory non-compliance. While the psychotropic register is not mandatory and decisions regarding what to include in the register is up to the organisation, each organisation maintained a psychotropic register and included within the register all drugs listed as a psychotropic in guidance published by the Aged Care Quality and Safety Commission (see Supplemental Material 7 for information about psychotropic register and psychotropic medications used in Australia). This list includes antidepressants, antipsychotics, anxiolytics, anticonvulsants, lithium carbonate, anti-dementia medication and opioids. Organisations employ considerable staff resources to maintain the psychotropic register [Care manager/Nurse/0403].

#### Facilitators to implement change

While regulation was the immediate driver for change, the ability of aged care organisations to implement change was enabled by *previous quality initiatives* [Care manager/Nurse/0404, Pharmacist/0206] and *well-established clinical governance processes* [Care manager/Nurse/0403 and 0406]. Experienced staff within each organisation could identify both organisational success stories and areas for improvement in relation to the appropriate use of psychotropic medication. Existing clinical governance processes within each organisation provided mechanisms for policy development as well as support for dissemination of information and feedback regarding practice change [Care manager/Nurse/0403 and 0406].

#### Barriers to implement change

Participants identified challenges in implementing policy change to both staff and visiting health providers. Some of the key barriers were inconsistent prescriber engagement [Nurse practitioner/0303], shifting responsibilities [Nurse practitioner/0303] and workload implications [General practitioner/0209].

### Aged care organisations are enhancing quality improvement systems for psychotropic medications (Theme 2)

Participants explored the features of the quality improvement systems implemented within the participating aged care organisations for ensuring appropriate use of psychotropic medications. Each organisation was actively working to enhance their processes in terms of the appropriate use of psychotropics. Theme 2 examines the quality improvement system described by participants with a particular focus on the strengths, opportunities, challenges and outcomes of the system. The framework for learning health systems guided the analysis. Table 3 summarises the key findings from participant interviews in terms of this framework. Example quotes for Theme 2 are presented in Table 4 and discussed below.

**Table 3.** Key findings from participant interviews aligned with the framework for learning health systems.

Components	Comments	Quotes
Core values	Participants expressed a strong focus on resident outcomes, adaptability, the need for innovation, accountability for outcomes and respect for scientific integrity and privacy. Leaders seek to recruit staff that share these values. Examples of cooperative and participatory leadership were provided.	Refer theme 2 (Strengths – Individual & organisational values)
Policy, legal, ethical pillar	Participating aged care organisations have the policy, legal and ethical systems in place to support practice as a learning health system. Data is routinely collected as part of national mandatory quality indicator reporting. Aged care organisations are continuing to develop and refine policy to ensure they meet current and expected regulatory requirements regarding psychotropics. Policy response within aged care organisations to changes in psychotropic regulations has focused on compliance. Participants recognised both the administrative burden of collecting data for compliance purposes and that there is an opportunity to better use this data to learn and communicate population/group-level findings.	Refer theme 1 (Implementation of psychotropic self-assessment tool)
Technological pillar	There has been work in each organisation to develop processes to efficiently and accurately collect data that can be input into the psychotropic register. All organisations use a mix of electronic and paper based/manual resources. Accurate recording of indication in line with organisation policy and regulation is pressure point for this process. How this is captured in systems and who is able to modify the record varies.	Refer theme 1 (Opportunities – Technological pillar)
Social pillar	Formal social structures differ with each organisation, but include: Advisory Board Meetings (2–3 times per year), Continuous Quality Improvement Meeting (once a month), Medicine Advisory Committee Meeting (3–4 times per year), Multidisciplinary Team meeting (case conference once a year with resident and family). These social structures provide opportunities for all stakeholders to meet and work through shared challenges.	Refer theme 1 (facilitators – clinical governance)
Scientific pillar	Participants provided examples of individuals and teams with the skills and capabilities to measure, analyse and develop and evaluate interventions in relation to use of psychotropics. These examples, however, tended to relate to specific people and projects rather than part of everyday business	Refer theme 1 (Facilitators – Previous quality initiatives)
Processes	Considerable attention placed on collecting practice data (‘practice to data’, P2D), ensuring the data is an accurate representation and some attention on interpreting the data in broad terms (‘data to knowledge’, D2 K) with regulatory focus. Participants recognised the need for a greater focus on ‘knowledge to practice, K2P’, especially in terms of balancing the need to meet regulatory compliance with increasingly sophisticated ways to improve care based on the data collected.	Refer theme 2 (Strengths – Organisational & individual values)
Outcomes	Clearer oversight of psychotropic use within the facility. A shift in individual and system defaults regarding use of psychotropics throughout the sector. A trend towards multidisciplinary team-based management of changed behaviours in residents with dementia. More involvement of residents, families and carers through requirements surrounding consent.	Refer theme 2 (Outcomes)

**Table 4.** Supporting quotes for theme 2 (Aged care organisations are enhancing quality improvement systems for psychotropic medications).

Theme 2 components	Supporting quotes
*Strengths*	
Individual & organisational values	*Clinical pharmacist/0208:* They are a very, very good organisation … when a new system is implemented, they’ll engage staff just to help implement that system. … when we have a [Medicines Advisory Committee (MAC)] meeting, … . and we really go through the figures. There is very clear planning by the organisation to reduce numbers and see, for example, ‘Okay. We’re going to reduce psychotropics, but how are we going to support the staff? What additional things do we need to do?’
	*Care manager/Nurse/0404:* The environment needs to be calm and friendly and homely feeling, so that's definitely here. However, where the environment is not possible, the staffing can itself create that magic in the sense – so we have recruited staff and then I also look – when we do the recruitment, we look in the behavioural qualities of the staff … And then we are looking at just moulding that behavioural component to step back basically.
	*Clinical governance/Nurse/0301:* And we’ve started and we’ve learnt and we’ve mucked up and we’re back again. And lessons learnt and we’re really invested in getting it right. We really want to have great outcomes for clients at the end of the day. And we want to be compliant.
Ethical, legal & policy support for data collection & use	*Nurse practitioner/Nurse/0204, Care manager/Nurse/0406:* Refer to theme 1 (Implementation of psychotropic self-assessment tool) quotes
Social structure supporting governance & quality improvement	*Care manager/Nurse/0403 and 0406:* Refer to theme 1 (Facilitators to implement change – Established clinical governance) quotes.
*Opportunities*	
Technological pillar	*Clinical governance/Nurse/0301:* So we use [a digital health system],. We've migrated to the cloud. All really boring, but important process stuff in terms of the monitoring of restrictive practice when they're in place. We had to wait for this upgrade to be able to access a much cleaner, smoother, kind of monitoring system. And so we're really hoping having a good look at our psychotropic management now to develop a process, embed the process, and do some change management to really reduce the number of forms and paperwork and meetings …
	*Clinical governance/Nurse/0402:* It'd be great to have a medication management system where all our polypharmacy reports, the psychotropic reports can just be populated and when a resident comes in, the pharmacy can be across, ‘Okay. These are the medicines.’ The GP *[General Practitioner]* can prescribe them. They're all there, ready to go. So the staff are really aware of all of that prior to having conversations with the family and the resident when they arrive. But we'll get there. I'm confident that having conversations with [our leadership team] now, hopefully, we'll get there.
	*Nurse practitioner/Nurse/0204:* Refer to theme 1 (psychotropic self-assessment tool) quotes.
Scientific pillar	*Care manager/Nurse/0404 and Pharmacist/0206:* Refer to theme 1 (facilitators – previous quality initiatives) quotes.
*Challenges*	
Balancing compliance & quality care	*Care manager/Nurse/0403:* Refer to theme 1 (psychotropic self-assessment tool) quotes.
	*Clinical pharmacist/0304:* Yeah, this is just across the scope. And the other anxiety being caused that the accreditation people are coming in and they're saying, send us this information on the percentage of people on psychotropics. And they're using that raw figure, they're saying you've got too many on them. And you just can't do that. It just doesn't relate at all. Basically, it's an individual basis that you make these decisions. It depends on their diagnosis. You can only make that comparison if they had identical diagnoses.
Effective interprofessional collaboration	*Care manager/Nurse/0202:* So we do have a process … So if the nonpharmacologic inventions doesn't work or the toolbox strategies wasn't effective, we escalate it to what we call a [behaviour support plan]. And that involves escalating it to the dementia consultant … . And they do review and just have a look whether there are other behaviour strategies that we've missed. And it's mainly more personalised then if we get family involved. Just mainly exploring on mental health history. If there's any history of trauma … So more on an interdisciplinary approach and a holistic approach.
	*Clinical governance/Nurse/0301:* And part of that's about the GP *[General Practitioner]* engagement and what role and what leadership role they can take in that. Because currently, it's a very, very passive role. So the duty of care is on us to keep pushing back to them. We'd really like to have a partnership approach with them to manage that because that would really assist with change to have engagement.
	*Clinical pharmacist/0504:* I think start involve the GP more in the [medication advisory committee] meetings as well and also try to organise to be on site on the same day as the GP. So that way, we've got a collaborative prescribing. Because I've been to a facility on the same day as a GP, and she's pretty much just asking me for advice on different patients, and we look at the blood test results together. We just look at the medication chart and see what sort of behaviour, what kind of issues they're facing and ask the nurse as well to give some input as well. So then come up with initiating someone with an antidepressant to help with constantly crying and agitated – so that's one of a good outcomes that I had recently.
*Outcomes*	
Use of psychotropic medications is reducing	*Clinical governance/Pharmacist/0503:* I've seen a huge reduction in use of psychotropics, especially the antipsychotics. If you put antidepressants in there, they just stay the same. They haven't really seen much difference there. But we've seen a huge decrease in Risperdal use. That used to be much higher. And a decrease in regular and PRN *[pro re nata or when required]* use. Benzos, well, off the top of my head, I think that has decreased as well, but the antipsychotics, definitely.
	*Dementia care/Occupational therapist/0205:* I think the best thing about this change has been that there is – it's very easy for people to prescribe medication, and then there isn't often a process to de-prescribe. So I think the best thing about this change has been putting in place processes that ensure that we are reviewing the psychotropic medication for cessation, and that they're not being put on this psychotropic for the rest of their life. So I think that that's probably been the best outcome.
	*Clinical governance/Nurse/0301:* * … *when the restrictive practices really became tighter. And I know within the first year, just by having a process we could really see the reduction. I think it was a 25% reduction in prescriptions for psychotropics.
Shared decision making is improving	*Embedded pharmacist/Pharmacist/0101:* And I have been dealing with family of residents. So once I’ve come up with my plan, I’ll contact the resident’s family just to notify them and just see their opinion. And they’re really happy with that just having a pharmacist on to have a chat to them and to explain to them what we’re doing, rather than just finding it out a bit later. … I just want to make sure that all the resident’s next of kin are all involved in this decision making.
	*Resident family member/Consumer/0210:* I can tell you from my point of view, in a risk versus benefit assessment for [my husband], the improvement to his quality of life and his calmness of demeanour and, generally, his happiness – I mean, obviously, I'm not him. I can't feel what he's feeling, but I am very close to him. And I have seen him, as I said, suffer from these problems for over a year. He is greatly – sorry, he benefits, to my mind, greatly from the medication. And the risk that he takes of an adverse cardiac or – I'm not sure what you'd call it – cerebral ischemic or haemorrhage event is worth it for the benefit.

Note: Identifier – Role/Profession/Organisation and Participant Code.

#### Strengths

The *individual and organisational values* expressed in interviews aligned well with core values needed to support quality improvement systems. Participants from a wide range of roles and professions were resident-focused, adaptable, open to innovation, respectful of individual privacy and looking for ways of working that were transparent and shared accountability for outcomes [Clinical pharmacist/0208, Care manager/Nurse/0404, Clinical governance/Nurse/0301]. The accounts of family members aligned with the descriptions of participating staff ‘[… I knew the system. I knew the people, and I knew how caring how they were and how good their numbers were according to the ratios between patient and carers’ [Resident family member/0211]. Aged care organisations had strengths in terms of the infrastructure needed for quality improvement systems. The *ethical, legal and policy support for collecting and using practice data* were well developed, especially in terms of collecting and reviewing data to meet existing regulatory requirements [Nurse practitioner/Nurse/0204, Care manager/Nurse/0406]. The *social structures supporting clinical governance* were also well developed within each organisation. Organisations used a variety of advisory boards, quality improvement and medication advisory committees to review compliance or other quality data [Care manager/Nurse/0403 and 0406].

#### Opportunities

Participants highlighted improvements that aged care organisations could make to further enhance the quality improvement systems for psychotropic medications. Opportunities included further developing technological and scientific pillars, as well the processes in place to collect, interpret and improve outcomes based on data.

##### Technological pillar

There was an opportunity to improve the accuracy and efficiency of data collected to maintain the psychotropic register for each organisation. None of the digital systems used by the age care organisations captured all data needed for completing the psychotropic register or behaviour support plans prior to introduction of the regulatory changes [Clinical governance/Nurse/0301]. Common challenges included the need to improve consistency of documentation regarding the indication for psychotropic medications, dates of review, and procedures for obtaining and documenting consent from the resident or their substitute decision-maker [Clinical governance/Nurse/0301 and 0402]. Each organisation had sought to implement both policy and technological solutions to address these challenges (although they differed in terms of stage of progress and the affordances their current system provides). Examples include organisations working closely with electronic medication management providers to collect key data for the psychotropic register at point of prescribing [Nurse practitioner/Nurse/0204], and making decisions regarding who can enter and amend the electronic recording of indication for a psychotropic medication ‘ … .every time when a GP prescribes a psychotropic medication or any changes happens in that prescription, it goes automatically into the online system’ [Nurse practitioner/Nurse/0204].

##### Scientific pillars

Participants tended to focus on individual clinical review or compliance requirements rather than completing continuous quality improvement cycles at the facility level. Quality improvement systems require processes for a continuous cycle of collecting practice data (‘practice-to-data’), analysing that data (‘data-to-knowledge’) and using that knowledge to improve care (‘knowledge-to-practice’). Each organisation had strong (and improving) processes regarding collecting practice data on the use of psychotropic medications as these processes were required for regulatory reporting. In terms of completing the quality improvement loop (creating knowledge on the basis of the data and using that knowledge to change practice); there were examples of individuals, teams and projects within each organisation completing quality improvement process on psychotropic medications [Care manager/Nurse/0404, Pharmacist/0206], but participants did not describe these steps as embedded within day-to-day processes. Enhancing the scientific capabilities of the organisation would mean that individuals, teams and committees would have the ability to measure and interpret key data and then develop and evaluate interventions to improve use of psychotropic medications.

#### Challenges

Two key challenges were identified in the participant interviews.

##### Balancing compliance and quality care

A frequent challenge expressed by participants was that perceived compliance requirements competed with efforts to provide high quality care. The primary concern was that (i) meeting formal and informal regulatory requirements took considerable work and (ii) that some of these requirements were not sufficiently targeted to improving care in individual residents. The set up and maintenance of a psychotropic register based on the *Psychotropic self-assessment tool* within each organisation was a commonly cited example of the challenge of balancing compliance and quality care [Care manager/Nurse/0403, Clinical pharmacist/0304]. The decision to maintain the psychotropic register for all medications with psychotropic effects and for all care recipients across the organisation has significant workload implications for the organisation and visiting health professionals. Aged care organisations felt the need to follow guidance provided regarding the *Psychotropic self-assessment tool* despite concerns among some participants that this approach would not appropriately target efforts to improve use of psychotropic medications in people living with dementia.

##### Effective interprofessional collaboration

Many participants identified that the rate and extent of change in regulation and practice regarding psychotropic medication use has created challenges for interprofessional collaborative care. Specific contributors include the shift in responsibilities regarding overall responsibility for ensuring appropriate use of psychotropics and differing interpretations regarding best-practice and/or appropriate ways to meet regulatory requirements. Staff of aged care homes identified that they were seeking ways to engage more productively with prescribers and other visiting health professionals to ensure a consistent approach [Clinical governance/Nurse/0301, Clinical pharmacist/0504]. In recognition of their responsibility for appropriate management of changed behaviours, some aged care organisations have implemented interdisciplinary team-based care for managing changed behaviour. One organisation had a long history of team-based care in this area, while another explicitly set out to build this capacity. Whether newly implemented or based on existing teams, each organisation was working through a process of change in relation to the specific roles and responsibilities of facility staff and those of visiting health professionals (especially prescribers) for managing changed behaviour in residents living with dementia [Care manager/Nurse/0202].

### Outcomes

Participants perceived the psychotropic register and behaviour support plans have provided organisations with a greater level of oversight. Many participants reported that regulatory and policy changes have changed practice in relation to use of psychotropic medications. Regulatory requirements and the need for improved documentation have triggered further discussions about specific prescribing indications for the psychotropic medications. Most participants reported that *psychotropic medications were less likely to be prescribed and more likely to be withdrawn* [Clinical governance/Pharmacist/0503, Dementia care/Occupational therapist/0205, Clinical governance/Nurse/0301]. Participants also discussed an *increased tendency to involve the person with dementia and family in treatment decision-making* [Embedded pharmacist/0101]. Family and caregivers noted *regular discussion regarding the risks and benefits of psychotropic medications* and confirmation of consent [Resident family member/0210].

## Discussion

Regulation has driven changes in processes regarding psychotropic medication use in Australian residential aged care homes. These changes have brought greater oversight and encouraged aged care organisations to adopt additional responsibilities for managing changed behaviour in dementia. The participating organisations had quality improvement systems supporting appropriate use of psychotropic medication and were working to enhance these systems in response to regulatory and practice change. Opportunities for further development were inter-related; building additional scientific capabilities to facilitate learning from practice data and implementing change as well as supporting, engaging and empowering interdisciplinary teams. Ongoing challenges include navigating the perceived tension between regulatory compliance and continuous quality improvement, and bringing the diverse range of stakeholders together to work toward shared goals.

Our findings align with a review on the impact of system-level interventions on psychotropic medication use in residential aged care homes (Langford et al., 2020) and the US experience following the US Nursing Home Reform Act (Briesacher et al., 2005; Hawes et al., 1997; Hughes & Lapane, 2005). Mandatory changes brought about through legislation, such as the Nursing Home Reform Act in the US, tend to have the greatest measurable impact on outcomes (e.g. use of psychotropic medications) (Langford et al., 2020). However, regulation is a blunt tool, and additional approaches are needed to support best care, such as clinical guidelines, audit and feedback and practitioner education (Hawes et al., 1997; Hughes & Lapane, 2005). Participants in our study recognised the benefits of regulation but also experienced challenges in implementing regulation with focus on quality. A common challenge was bringing together internal and external stakeholders to formulate clinical governance processes. A strong commitment to ensuring regulatory compliance and perceptions within the sector regarding appropriate implementation of the regulations have been influential in determining a clinical governance system that goes beyond the explicit regulatory requirements. Staff of each aged care organisation expressed concern regarding whether changes implemented in response to new regulations were optimal for improving resident outcomes but also perceived they were unable to modify these regulations.

Day to day processes within aged care homes tended to focus on compliance or individual clinical care rather than learning from data and implementing targeted change processes. Each organisation had, or was building, interdisciplinary teams to manage change behaviour in residents living with dementia, but each organisation experienced challenges in bringing together the multidisciplinary team, especially visiting health professionals such as prescribers and pharmacists. There is increasing recognition of the value of routinely collected aged care data for informing clinical practice (Seaman et al., 2021). In the context of this study, a learning health system framework helped to describe the people, processes and infrastructure supporting the appropriate use of psychotropic medications. The framework also helped identify components of the current quality improvement process that would benefit from further attention and provides a model for addressing current challenges.

The study highlighted the need for building capacity and processes to meaningfully interpret clinical data to drive practice change. Building greater capacity for collecting, interpreting and responding to clinical data on psychotropic medication use will require developing skills in data science and quality use of medications. Implementing new electronic information systems as recommended by the Royal Commission into Aged Care Quality and Safety will provide greater opportunities for using clinical data for quality improvement (Seaman et al., 2021). Our findings were consistent with the identified need for better use of data to identify and respond to emerging quality use of medications issues at the organisational level (Picton et al., 2020). These data can be used to design, implement and evaluate local strategies to improve care. Our study suggests that considerable steps are being made towards achieving this within existing clinical governance structures. However, an ongoing challenge is bringing isolated quality improvement projects into the day-to-day activities of clinical governance.

Building interdisciplinary teams with this capacity and focus which include internal and visiting health professionals also provides a way to address some of the challenges identified by participants. Specifically, it provides a mechanism to bring the team together to develop shared goals in relation to psychotropic medications use and review, and discuss local policy in response to regulatory requirements. This is also an important role of multidisciplinary Medication Advisory Committees as set out in new guidance for aged care organisations published by the Australian Government Department of Health and Aged Care (Australian Government, 2022b). Once functioning, these processes could inform efforts to better link compliance and quality activities (Cross et al., 2025). Well-functioning quality improvement processes could provide organisations with principled ways to modify compliance processes with a view to ensuring improved outcomes.

### Strengths and limitations

The study obtained the perspectives of a diverse range of people involved in psychotropic medications management in four aged care organisations located in different states in Australia. The timing of the study, 18 months after large-scale regulatory changes in relation to psychotropic medications management, helped to capture quality improvement systems within organisations as they approach a new steady-state where they work to fine-tune the system and address challenges. While it was good to include some family members and residents, a different study would be needed to comprehensively explore the lived experience of residents living with dementia and their family and informal caregivers. Similarly, the ability to explore differences between professional groups in relation to quality use of psychotropics is limited by the small numbers of participants in each group, especially among prescribers and allied health practitioners (e.g. physiotherapists, social workers).

## Conclusions

A remarkable amount of change has occurred in policies, procedures and infrastructure regarding psychotropic medication management in each of the participating organisations following recent regulatory changes. These changes have impacted the way aged care organisations, facilities and individual health practitioners use psychotropic medicines. Key opportunities for improving use of psychotropic medications within aged care organisations include improving the capacity to use local data to improve care and building interdisciplinary teams to facilitate collaborative care.

## Supplementary Material

Supplemental Material 3

Supplemental Material 6

Supplemental Material 7

Supplemental Material 4

Supplemental Material 5

Supplemental Material 1

Supplemental Material 2

## Data Availability

All data analysed during this study are included in this published article.
