# Supplementary material for: Exploring quality improvement processes for psychotropic medication use in Australian residential aged care homes: a qualitative study
Source: J Pharm Policy Pract. 2025 Sep 22;18(1):2557873. doi: 10.1080/20523211.2025.2557873 (PMC12456038; doi:10.1080/20523211.2025.2557873)
Supplement: Supplemental Material 3 [file JPPP_A_2557873_SM1101.docx]

# **Additional file 3 – Geographical location of participating aged care organisations**

| **Geographical location** | **MMM Category** | **State** | **Total facilities** | **Average bed size** |
| --- | --- | --- | --- | --- |
| Metropolitan area | MM1 | QLD | 3 | 79 |
|  |  | WA | 10 | 76 |
|  |  | VIC | 4 | 55 |
|  |  | NSW | 3 | 220 |
| Regional centre | MM2 | QLD | 3 | 89 |

**Abbreviation:** MMM, Modified Monash Model; NSW, New South Wales; QLD, Queensland; VIC, Victoria; WA, Western Australia

**Note:**

- There were four participating aged care organisations – one from each state.
- MMM 2019 consists of 7 categories – MM1 (Metropolitan area), MM2 (Regional centres), MM3 (Large rural towns), MM4 (Medium rural towns), MM5 (Small rural towns), MM6 (Remote communities), MM7 (Very remote communities). For more details on MMM 2019 see <https://www.health.gov.au/topics/rural-health-workforce/classifications/mmm?language=und>
