# Supplementary material for: Exploring quality improvement processes for psychotropic medication use in Australian residential aged care homes: a qualitative study
Source: J Pharm Policy Pract. 2025 Sep 22;18(1):2557873. doi: 10.1080/20523211.2025.2557873 (PMC12456038; doi:10.1080/20523211.2025.2557873)
Supplement: Supplemental Material 6 [file JPPP_A_2557873_SM1100.docx]

# **Additional file 6 – Coding Scheme representing deductive and inductive codes**

| Deductive codes for Learning Health System Framework | |  | Inductive codes | |
| --- | --- | --- | --- | --- |
| *Component* | ***Code*** |  | ***Code group*** | ***Code*** |
| Core Values | Adaptability |  | Structure and context | Quality improvement |
|  | Cooperative and participatory leadership |  |  | Administrative burden |
|  | Equity |  |  | Balancing compliance and care |
|  | Inclusiveness |  |  | Barrier |
|  | Open innovation |  |  | Clinical governance |
|  | Privacy |  |  | Managing change |
|  | Resident focused |  | Psychotropic medication use | Workforce |
|  | Scientific integrity |  |  | Definition |
|  | Shared accountability |  |  | Adverse event |
|  | Solidarity |  |  | Chemical restraint |
|  | Transparency |  |  | Behaviour support plan |
| Pillars | Ethical |  |  | Culture |
|  | Legal |  | Interprofessional practice and communication | Psychotropic register |
|  | Policy |  |  | Responsibility |
|  | Scientific |  | Consumer experience | Team roles |
|  | Social |  |  | Lived experience |
|  | Technological |  |  | Medicines information |
| Processes | Data to knowledge (D2K) |  |  | |
|  | Knowledge to practice (K2P) |  |  |  |
|  | Practice to data (P2D) |  |  |  |
| Outcomes | Healthcare costs |  |  |  |
|  | Population health |  |  |  |
|  | Provider experience |  |  |  |
|  | Resident experience |  |  |  |
