# Supplementary material for: Exploring quality improvement processes for psychotropic medication use in Australian residential aged care homes: a qualitative study
Source: J Pharm Policy Pract. 2025 Sep 22;18(1):2557873. doi: 10.1080/20523211.2025.2557873 (PMC12456038; doi:10.1080/20523211.2025.2557873)
Supplement: Supplemental Material 7 [file JPPP_A_2557873_SM1099.docx]

## **Additional file 7 – Psychotropic self-assessment tool (Psychtropic register)**

The psychotropic self-assessment tool supports aged care service provider organisations to document the use of psychotropic medication(s) for all residents, including as needed medications. Following are key resources for psychotropic self-assessment tool:

- Information for aged care about the psychotropic medications used in Australia is available at <https://www.agedcarequality.gov.au/sites/default/files/media/acqsc_psychotropic_medications_v11.pdf>
- The psychotropic medications self-assessment tool template is available at <https://www.agedcarequality.gov.au/sites/default/files/media/psychotropic-medications-self-assessment-tool.docx>
- Frequently asked questions about psychotropic self-assessment tool is available at <https://www.agedcarequality.gov.au/sites/default/files/media/frequently-asked-questions-psychotropic-self-assessment-tool.pdf>
