# Supplementary material for: Exploring quality improvement processes for psychotropic medication use in Australian residential aged care homes: a qualitative study
Source: J Pharm Policy Pract. 2025 Sep 22;18(1):2557873. doi: 10.1080/20523211.2025.2557873 (PMC12456038; doi:10.1080/20523211.2025.2557873)
Supplement: Supplemental Material 4 [file JPPP_A_2557873_SM1098.docx]

## **Additional file 4 – Recruitment**

Proposed stakeholder groups for semi-structured interviews:

- Facility manager,
- Clinical care and /or quality manager,
- Registered nurse,
- Enrolled nurse,
- Personal care attendant/Community care worker,
- General practitioner,
- Pharmacist - dispensing,
- Pharmacist - medication review/QUM,
- Additional prescribers - geriatricians, practice nurses, others,
- Resident,
- Family and other informal carers
