# Supplementary material for: Exploring quality improvement processes for psychotropic medication use in Australian residential aged care homes: a qualitative study
Source: J Pharm Policy Pract. 2025 Sep 22;18(1):2557873. doi: 10.1080/20523211.2025.2557873 (PMC12456038; doi:10.1080/20523211.2025.2557873)
Supplement: Supplemental Material 1 [file JPPP_A_2557873_SM1096.docx]

## **Additional file 1 – Regulatory changes**

Changes to the Quality of Care Principles 2014^1^ came into effect July 2021.^2^ The key changes were clearer definitions of restrictive practices, including chemical restraint, and the requirement to document specific details regarding chemical restraint in a care recipient’s behaviour support plan. These changes came with guidance to aged care organisations emphasising their overall responsibility for ensuring compliance, and a Psychotropic self-assessment tool.^3^ The Psychotropic self-assessment tool guided organisations to collate data on the use of psychotropic medicines, including indication, details regarding informed consent, whether the psychotropic is used as chemical restraint, whether a behaviour support plan is in place, monitoring for effectiveness and harms, and date of last review.

## **Definition of “chemical restraint” and documentation in a behaviour support plan^1^**

Part 4A of the Quality of Care Principles 2014 deals about behavioural support and restrictive practices in residential care.

***Chemical restraint:*** Part 4A Division 2 deals with restrictive practices, under which 15E deals on practices or interventions that are restrictive. Chemical restraint is defined under 15E(2) as follows:

(2) Chemical restraint is a practice or intervention that is, or that involves, the use of medication or a chemical substance for the primary purpose of influencing a care recipient’s behaviour, but does not include the use of medication prescribed for:

1. the treatment of, or to enable treatment of, the care recipient for:
   1. a diagnosed mental disorder; or
   2. a physical illness; or
   3. a physical condition; or
2. end of life care for the care recipient.

***Documentation in the behavioural support plan:*** Division 3 of Part 4A deals about circumstances for the use of restrictive practices. 15FC enlists the additional requirements for the use of restrictive practices that are chemical restraint. With regards to the behavioural support plan 15FC(1(b)) states as follows:

1. the following matters have been documented in the behaviour support plan for the care recipient:
   1. the assessments;
   2. the practitioner’s decision to use the chemical restraint;
   3. the care recipient’s behaviours that are relevant to the need for the chemical restraint;
   4. the reasons the chemical restraint is necessary;
   5. the information (if any) provided to the practitioner that informed the decision to prescribe the medication for the purpose of using the chemical restraint; (va) that the approved provider is satisfied that the practitioner obtained informed consent to the prescribing of the medication; (vb) the details of the prescription for the prescribed medication, including its name, dosage and when it may be used;
   6. a description of any engagement with persons other than the practitioner in relation to the use of the chemical restraint;
   7. a description of any engagement with external support services (for example, dementia support specialists) in relation to the assessments;

**References**

1. Australian Government. Quality of Care Principles 2014. 2024 [Available from: <https://www.legislation.gov.au/F2014L00830/latest/text>
2. Australian Government. Restrictive practices in aged care – a last resort. 2024 [Available from: <https://www.health.gov.au/topics/aged-care/providing-aged-care-services/training-and-guidance/restrictive-practices-in-aged-care-a-last-resort#:~:text=On%201%20July%202021%2C%20amendments,approved)%20residential%20aged%20care%20providers>
3. Aged Care Quality and Safety Commission (ACQSC). Psychotropic self-assessment tool. 2022; [Available from: <https://www.agedcarequality.gov.au/resources/self-assessment-tool-psychotropic-medications>
